# Discordant phenotype caused by *TREX1* variant in siblings with Aicardi-Goutières syndrome

**DOI:** 10.1186/s12969-025-01168-2

**Published:** 2025-11-05

**Authors:** Rou Liu, Stefanie Kretschmer, Paulina Switala, Mohamed Attia, Min Ae Lee-Kirsch, Christine Wolf

**Affiliations:** 1 Department of Pediatrics, Medizinische Fakultät Carl Gustav Carus, Technische Universität Dresden, Dresden, Germany; 2 Vinzentius Krankenhaus Landau, Landau, Germany; 3 German Center for Child and Adolescent Health (DZKJ), Partner Site Leipzig/Dresden, Dresden, Germany

**Keywords:** Aicardi-Goutières syndrome, Familial chilblain lupus, TREX1, Type I interferonopathy, Interferon, Genetics

## Abstract

**Background:**

Autosomal recessive Aicardi-Goutières syndrome (AGS) and autosomal dominant familial chilblain lupus (FCL) are rare type I interferonopathies that can both result from loss-of-function variants in the *TREX1* gene, which encodes a DNA exonuclease. Although phenotypic variability is well recognized in *TREX1*-related disorders, intrafamilial phenotypic discordance is seldom seen.

**Case presentation:**

We describe two siblings carrying a novel homozygous *TREX1* variant (c.341G > T, p.Arg114Leu) who exhibit strikingly different clinical phenotypes. The younger sibling presented at 4 months of age with features of AGS, including tetraspasticity, muscular hypotonia and global developmental delay. Brain MRI showed brain atrophy and white matter abnormalities. In contrast, his older brother developed cutaneous chilblain lesions during the cold season at age 3 but was otherwise normally developed. Despite these divergent clinical presentations, both children demonstrated a highly elevated interferon signature.

**Conclusions:**

This case report expands the genetic and clinical spectrum of *TREX1*-related disorders and illustrates the considerable phenotypic variability associated with biallelic *TREX1* variants in AGS. It highlights the complexity of genotype-phenotype correlations in type I interferonopathies and underscores the need for further research into factors that modulate disease expression.

## Background

Aicardi-Goutières syndrome (AGS, OMIM #225750) is a rare inherited autoinflammatory disorder that primarily manifests in infancy and is characterized by progressive encephalopathy, intracranial calcifications, and chronic cerebrospinal fluid (CSF) lymphocytosis [1]. In its classic form, AGS typically presents within the first months of life, often following an initial period of apparently normal development. Hallmark features include subacute encephalopathy with irritability, persistent crying, feeding difficulties, and psychomotor regression or delay. Affected individuals frequently develop dystonia, spasticity, and truncal hypotonia, with some experiencing seizures, elevated liver enzymes, and episodes of unexplained sterile pyrexia. Approximately 20% of patients develop cold-induced inflammatory skin lesions, also referred to as chilblains [2]. The clinical picture of AGS closely mimics that of congenital viral infections, despite absence of a detectable infectious agent. Other organ systems, such as the eyes, thyroid, and hematological system are variably involved [3]. Among the nine genes implicated in AGS, biallelic variants in *TREX1* are responsible for AGS type 1 [4].

Familial chilblain lupus (FCL, OMIM #610448) is a rare autosomal dominant cutaneous form of lupus erythematosus caused by heterozygous *TREX1* variants [5–7]. It typically presents in early childhood with painful, bluish-red lesions affecting the acral areas, such as fingers, toes, and ears, most commonly during the cold season. Some individuals with FCL also develop antinuclear autoantibodies or experience arthralgias [5]. Unlike AGS, patients with FCL do not show central nervous system involvement [5].

The *TREX1* gene encodes the three prime repair exonuclease 1, a DNase essential for degrading single-stranded DNA byproducts generated during DNA repair or reverse transcription of retroelements [8]. This process prevents intracellular accumulation of immunostimulatory DNA. Loss-of-function variants in *TREX1*, as identified in patients with AGS and FCL, impair DNA clearance and result in chronic activation of type I interferons (IFN) via the DNA sensing cGAS-STING pathway [8]. While type I IFNs are essential for host defense against pathogens, uncontrolled, excessive type I IFN signaling can be harmful, driving autoinflammation and autoimmunity. The therapeutic benefit of Janus kinase (JAK) inhibitors in both AGS and FCL further highlights the central role of type I IFN dysregulation in the pathogenesis of these diseases [9].

Here, we report a novel homozygous *TREX1* variant in two siblings with discordant clinical presentation, expanding the clinical and genetic spectrum of *TREX1*-related diseases. Our findings support the concept that AGS, FCL and other *TREX1*-related disorders exist on a shared clinical spectrum, rather than as completely distinct disease entities.

## Case presentation

### Case 1

**Fig. 1:**
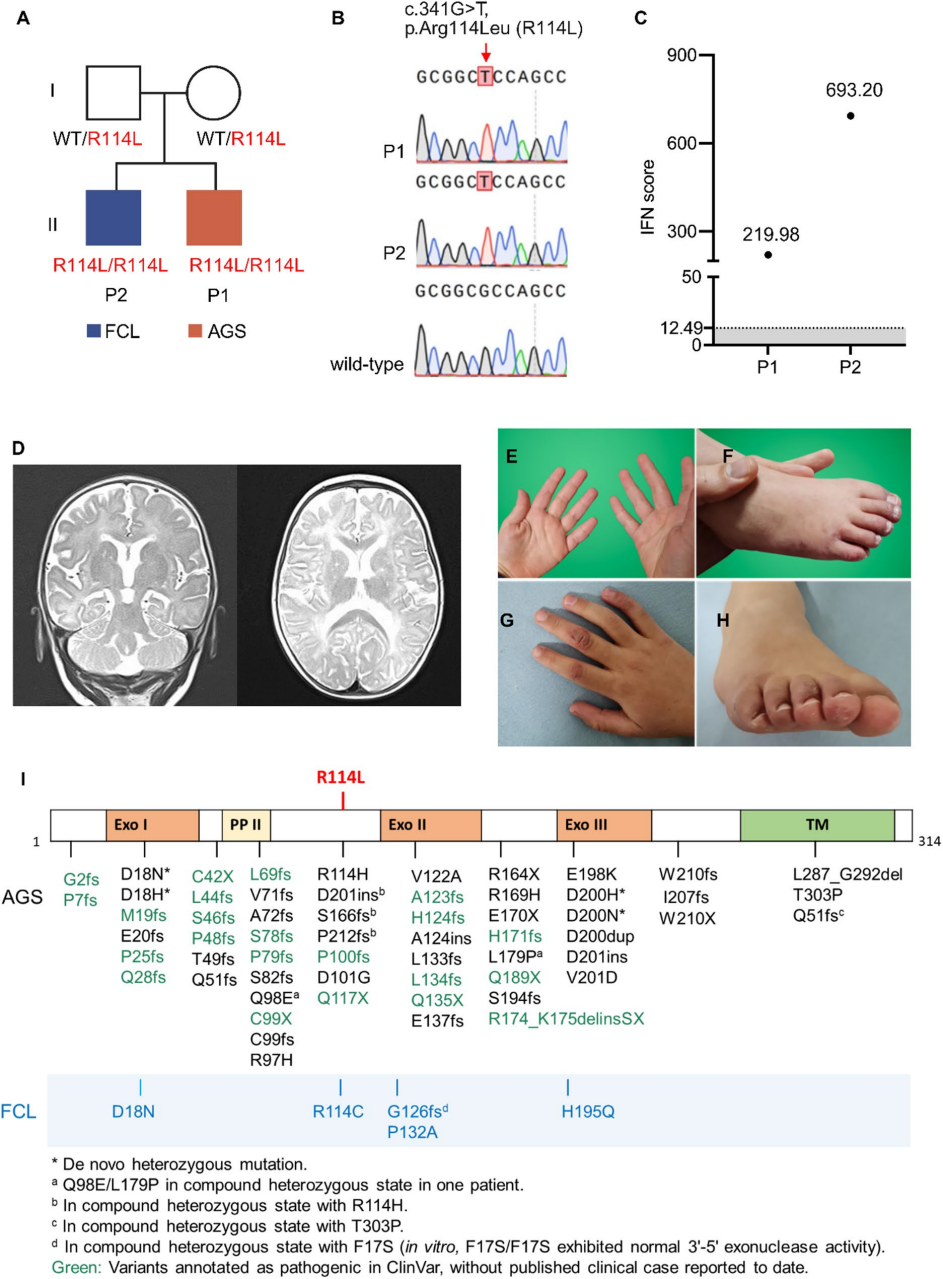
Family pedigree, electropherograms, clinical data and schematic of the TREX1 protein. (**A**) Pedigree chart depicting patients 1 and 2 (P1, P2) and their variant status. (**B**) Electropherograms showing the homozygous c.341G > T variant in both siblings. (**C**) Interferon scores of both patients. The grey area indicates the normal range < 12.49. (**D**) Brain MRI imaging of P1. T2-weighted coronal view (left) and T2-FLAIR transversal view (right), showing white matter lesions and signs of cortical atrophy. (**E**-**H**) Chilblain lesions in P2 at 4 years (E, F) and at an age of 7 years (**G**, **H**). **I.** Schematic illustration of the TREX1 protein including domains and previously described AGS and FCL variants in black and blue, respectively. Exo, exonuclease domain; PII, polyproline II motif; TM, transmembrane helix domain. The R114L variant identified in this study is shown in red

Patient 1 is a 16 months-old male born at 36 + 2 weeks of gestation to healthy consanguineous parents of Moroccan origin. He was small for gestational age but otherwise unremarkable at birth (Fig. 1A). At 4 months, he developed increasing irritability, muscular hypotonia with poor head control, spasticity affecting all extremities, and feeding difficulties including dysphagia and gastroesophageal reflux. No skin lesions were noted. Laboratory investigations were largely unremarkable apart from mildly elevated liver enzymes. Thyroid function was normal, and extensive infectious screening was non-contributory. Anti-CMV IgG (> 200, normal < 16) and IgM (1.6, normal < 0.8) levels were elevated, but these titers declined over time, and a CMV-DNA PCR was negative, consistent with a recent, but not congenital, CMV infection. Echocardiography, chest X-ray, external sleep EEG, and ophthalmologic assessment were all within normal limits. Brain MRI demonstrated cortical thinning, enlarged ventricular and suprarachnoid spaces, T2 hyperintensities in the white matter of the bioccipital area, and abnormal signal in the thalamus, suggestive of an underlying neuroinflammatory or neurometabolic disorder (Fig. 1D).

Whole exome sequencing identified a previously unreported homozygous variant in the *TREX1* gene (c.341G > T, p.Arg114Leu; NM_033629.6), which is absent from the gnomAD database (Fig. 1B). Both healthy parents were heterozygous carriers of the p.Arg114Leu variant. In light of the clinical presentation and the known association of *TREX1* variants with AGS, the patient underwent interferon signature analysis. Quantitative RT-PCR of patient blood revealed markedly elevated expression of IFN-simulated genes, with an IFN score of 219.98 (normal < 12.49) (Fig. 1C, P1), strongly supporting the diagnosis of AGS.

The patient was started on the JAK inhibitor ruxolitinib (orally, 0.5 mg/kg/day divided into two doses). Over a 12-months follow-up period, the patient has demonstrated reduced irritability and improvements in developmental milestones, including sitting with support and enhanced emotional responses, despite continued delays.

### Case 2

Patient 2 is the 6-year-old brother of patient 1, who began developing mild chilblain lesions at the age of 3, typically during the cold season. The skin manifestations appeared as erythematous patches or nodules on the fingers and toes, which occasionally ulcerated and healed with hyperpigmentation (Fig. 1E-H). Aside from the skin findings, his physical examination, including comprehensive neurodevelopmental assessment, and laboratory tests were unremarkable. The chilblain lesions were managed with topical steroids. Genetic analysis confirmed that patient 2 harbored the same homozygous *TREX1* variant as his younger brother (Fig. 1B). Subsequent interferon signature analysis demonstrated a markedly elevated IFN score of 693.20 (Fig. 1C, P2), consistent with strong systemic type I IFN activation.

## Discussion and conclusions

We report two siblings with a novel homozygous *TREX1* variant (c.341G >T, p.Arg114Leu) who exhibited strikingly different clinical phenotypes. The younger sibling, patient 1, presented with signs of classical AGS, whereas the older sibling exhibited only mild chilblain lupus. Notably, a different amino acid substitution at the same residue - p.Arg114His - represents the most common TREX1 variant reported in AGS patients to date across diverse ethnic backgrounds. An overview of previously reported *TREX1* variants associated with AGS and FCL is provided in Fig. 1I [4, 6, 9–16].

TREX1 functions as a homodimer, with the amino acid residue Arg114 located at the dimerization interface, stabilizing the protein-protein interface [17]. The p.Arg114His variant disrupts dimerization and abolishes enzymatic activity, leading to cytosolic DNA accumulation with chronic type I IFN activation [8]. Thus, it is possible that the p.Arg114Leu substitution may exert a similar loss of function.

Notably, *TREX1* variants associated with FCL, such as p.Asp18Asn, are considered distinct in that they typically affect residues within the catalytic Exo domains (Fig. 1) [14, 18, 19]. Interestingly, the heterozygous *TREX1* p.Asp18Asn variant has been reported in families where some members were diagnosed with AGS and some with FCL [14, 19]. Furthermore, a homozygous p.Arg114Cys variant has been described in siblings with discordant phenotypes – one with FCL and the other with neuroinflammation [15] – mirroring the phenotypic variability in our patients.

While clinical non-penetrance is well recognized in *IFIH1*-related AGS and has been sporadically reported in *RNASEH2B*-related AGS [3], biallelic *TREX1* variants are typically associated with severe presentations of AGS, often with neonatal onset and profound disability [3]. However, our cases challenge this paradigm, demonstrating that the same homozygous *TREX1* variant can result in markedly different clinical presentations within the same family. This highlights the occurrence of variable expressivity in *TREX1*-related diseases, which may be influenced by modifying genetic or environmental factors, such as viral infections. Notably, patient 1 tested positive for CMV IgM, consistent with a recent CMV infection. Although congenital CMV infection was excluded, it is possible that exposure to viral DNA may have triggered dysregulation of the cGAS-STING pathway, precipitating or exacerbating the onset of severe AGS in this patient.

Consistent with the correlation between disease severity and the degree of type I IFN activation, AGS patients typically exhibit highly elevated IFN signatures in blood, whereas FCL patients generally display milder IFN activation [9]. Our mildly affected patient 2 showed a higher blood IFN score than his severely affected brother, patient 1. However, as patient 1 had been receiving JAK inhibitor therapy for 4 months at the time of IFN signature analysis, this discrepancy likely reflects the therapeutic impact of treatment.

*TREX1*-related AGS usually arises from biallelic variants, while FCL is inherited in an autosomal dominant manner. Nevertheless, both conditions share a pathogenic mechanism centered on TREX1 dysfunction, resulting in the overproduction of type I IFN. In line with this, our findings further support that AGS and FCL are not entirely distinct entities but exist on a spectrum of overlapping phenotypes. The observed intrafamilial phenotypic variability likely reflects the contribution of additional genetic and environmental modifiers to disease expression. A deeper understanding of these factors will improve diagnostic precision and inform personalized treatment strategies. Importantly, our findings underscore the necessity for thorough genetic counseling and long-term follow-up, even in apparently mildly affected individuals.

## Data Availability

All data generated or analysed during this study are included in this published article.

## Abbreviations

AGS: Aicardi-Goutières syndrome
CMV: Cytomegalovirus
CNS: Central nervous system
cGAS: Cyclic GMP-AMP synthase
dsDNA: Double-stranded DNA
FCL: Familial chilblain lupus
IFN: Interferon
JAK: Janus kinase
RT-PCR: Reverse Transcription Polymerase Chain Reaction
ssDNA: Single-stranded DNA
SLE: Systemic lupus erythematosus
STING: Stimulator of interferon genes
TREX1 3’-repair: Exonuclease 1
